# Bio-Active Products from Mangrove Ecosystems

**DOI:** 10.3390/md21040239

**Published:** 2023-04-14

**Authors:** Wenhan Lin, Guoqiang Li, Jing Xu

**Affiliations:** 1 State Key Laboratory of Natural and Biomimetic Drugs, Institute of Ocean Research, Peking University, Beijing 100191, China; 2 Key Laboratory of Marine Drugs, Chinese Ministry of Education, School of Medicine and Pharmacy, Ocean University of China, Qingdao 266003, China; 3 Collaborative Innovation Center of Ecological Civilization, School of Chemical Engineering and Technology, Hainan University, Haikou 570228, China

Mangrove communities represent the coastal habitats located in intertidal zones or brackish waters of tropical and subtropical coastal areas in over 118 countries [1]. Typical mangrove ecosystems including highly saline or brackish water, high solar irradiation, and tidal gradients, inducing mangrove residents to evolve biodiversity for environmental adaptations. Mangrove plants and the associated microorganisms encode unique biosynthetic genes which have the potential to generate chemical diversity and novelty with promising pharmaceutical applications. In recent decades, numerous metabolites with uncommon structures and efficacious bioactivities have been discovered from mangrove hosts and their associated microorganisms.

The emergence of drug-resistance and rising pathogen mutations attenuates the potency of current antimicrobial drugs. Thus, mining structurally novel and pharmacologically potent natural products from mangrove ecosystems has attracted a great deal of attention from chemists and pharmacologists. Additionally, a broad range of chemical structures with unique scaffolds imply that their biogenesis involves novel functional genes and corresponding enzymes with unique catalytic functions.

This Special Issue “Bio-Active Products from Mangrove Ecosystems” (https://www.mdpi.com/journal/marinedrugs/special_issues/mangrove_ecosystems, accessed on 3 March 2022) contains ten peer-reviewed articles, including three comprehensive reviews and seven research papers on different topics related to new approaches and information on bioactive compounds with privileged scaffolds, such as polyketides, alkaloids and terpenoids, derived from mangrove fungi with potential for anticancer, antimicrobial, antioxidant, antidiabetic, and immunosuppressive activities, and elucidation of ecological defense functions. Herein, we introduce a brief overview of the main achievements contributed by each study.

A review by Wu et al. [2] introduces unusual secondary metabolites from mangrove ecosystems, reporting 134 metabolites, which are classified into two major families in terms of the biological sources and 15 subfamilies according to the chemical structures, covering the period from 2010 to 2022. The structures, biological activities, biosynthesis, and total chemical syntheses of some unique compounds are included. A majority of these compounds are produced by mangrove-associated microorganisms, and more than 70% were isolated from endophyte fungi, indicating a remarkable chemical diversity of the microbial community. In addition, these compounds display diverse and remarkable biological activities and are frequently reported as antimicrobial and cytotoxic compounds. Structurally diverse secondary metabolites play a crucial role in the discovery of new natural products with interesting pharmacophores. Secondary metabolites featuring new scaffolds, unique ring systems, or unusual functional groups might merit the attention of chemists and biologists and could be a source of fresh pharmacophores with biological activity for the creation of drugs based on natural products.

The second review by Sulaiman et al. [3] provides a comprehensive review of antibacterial, antifungal, and antiviral natural products from the mangrove plants in Asia and the Pacific reported from 1968 to 2022. Among the 286 plant species, 119 exhibited antimicrobial effects, and a total of 114 antimicrobial natural products have been identified including 12 with MIC values below 1 µg/mL. A vast array of antimicrobial secondary metabolites that could be further examined for development of anti-infectives to mitigate infectious diseases such as the White Spot Syndrome Virus infection in aquaculture is described. In parallel, the use and promotion of mangrove plants in aquacultures are beneficial as the rise in the global population which requires a huge supply shrimps, prawns, crabs, and fish globally necessitates the preservation of mangroves.

Small molecules with different mechanisms of fibrinolysis action are desired for new antithrombotics and thrombolytics. Hang et al. [4] list a series of bioactive staplabin congeners which not only possess fibrinolytic activity but also exhibit various effects, such as anti-inflammatory, neuroprotective, and anti-cancer properties. The authors focused on the diverse biological activities of SMTP-7 (compound 1, also known as FGFC1), an isoindolone alkaloid from the marine fungi *Starchbotrys longispora* FG216 and *Stachybotrys microspora* IFO 30018, that possesses diverse bioactivities such as thrombolytic, anti-inflammatory, and anti-oxidative properties and selective anti-cancer activity. These properties make SMTP-7 an attractive option for the development of drugs for the treatment of various diseases, including cerebral infarction, stroke, ischemia/reperfusion damage, acute kidney injury, non-small cell lung cancer, and especially for cerebral infarction. The recent progress in structure–function relationships, preparation methods, identification of diverse biological activities and mechanism of SMTP-7 and its congeners is summarized, thereby illustrating its high therapeutic potential.

Cai et al. [5] reported 6 novel isocoumarins, namely talaromarins A-F, along with 17 known analogues from the mangrove-derived fungus *Talaromyces flavus* TGGP35. Their structures were elucidated by extensive analysis of spectroscopic data, modified Mosher’s method, and ECD spectra. Eleven compounds (including talaromarin F) showed similar or better antioxidant activity compared with the positive control trolox, of which 6,8-dihydroxy-3-(2-hydroxypropyl)-7-methyl-1*H*-isochromen-1-one was the most active and showed ABTS radical scavenging capacity with an IC_50_ value of 0.009 mM. Moreover, 5,6-dihydroxy-3-(4-hydroxypentyl)-isochroman-1-one, peniciisocoumarin D, penicimarin N, and pestalotiorin exhibited strong inhibitory activity against α-glucosidase with IC_50_ values ranging from 0.10 to 0.62 mM, while the positive control acarbose showed an IC_50_ value of 0.5 mM.

Four previously unreported drimane sesquiterpenoids, named ustusol F, 9-deoxyustusol F, ustusol G, ustusolate H, in addition to ustusolate I, ustusolate J, and ustusol B, were isolated and structurally characterized from the fermentation broth of the mangrove-derived *Aspergillus ustus* 094102 by Gui et al. [6]. Ustusolate I has two pairs of enantiomers of the gem diol in the side chain, namely (2*E*, 4*E*; 6,7-erythro)-ustusolate I and (2*E*, 4*E*; *ent*-6,7-*erythro*)-ustusolate I, and (2*E*, 4E, 6*R*, 7*R*)- ustusolate I and (2*E*, 4*E*, 6*S*, 7*S*)-ustusolate I, which were purified using HPLC. The antiproliferative activities of the isolated compounds were evaluated against 29 human cancer cell lines and a non-cancer cell line, of which ustusolate I showed an antiproliferative effect against the human tumor cells CAL-62 and MG-63 with IC_50_ values of 16.3 and 10.1 µM, respectively.

Wang et al. [7] first systematically evaluated the diversity of cultivable fungi associated with the medicinal mangrove *Acanthus ilicifolius* collected from the South China Sea. A total of 102 fungal strains were isolated from *A. ilicifolius* and 84 independent culturable isolates were identified using a combination of morphological characteristics and internal transcribed spacer (ITS) sequence analyses, of which 37 strains were selected for phylogenetic analysis. The identified fungi belonged to 22 genera within seven taxonomic orders of one phyla, of which four genera *Verticillium*, *Neocosmospora*, *Valsa*, and *Pyrenochaeta* were first isolated from mangroves. Thirty-one strains of fungi displayed strong cytotoxicity to different human cancer cell lines: A-549, HeLa, HepG, and Jurkat. Integrating a cytotoxic activity-guided strategy and fingerprint analysis, a well-known natural Golgi-disruptor and Arf-GEF inhibitor, brefeldin A, was quickly isolated from the active strains.

Feng et al. [8] reported three previously undescribed cytochalasins, namely phomoparagins A-C, together with five previously reported analogs from the mangrove-derived endophytic fungus *Phomopsis asparagi* DHS-48. Notably, phomoparagin A possessed an unprecedented 5/6/5/8/5-fused pentacyclic skeleton. These compounds were tested for their inhibitory activity against concanavalin A (ConA)/lipopolysaccharide (LPS)-induced spleen lymphocyte proliferation and the calcineurin (CN) enzyme. Phomoparagin B, phomopchalasins A and B, as well as cytochalasin H exhibited robust inhibitory activities with a relatively low toxicity. Moreover, phomoparagin B was shown to inhibit ConA-stimulated activation of NFAT1 dephosphorylation and block NFAT1 translocation in vitro, subsequently inhibiting the transcription of interleukin-2 (IL-2), whereas it directly inhibited calcineurin and did not require a matchmaker protein, such as the clinical immunosuppressants CsA and FK506.

Epigenetic manipulation was described as an effective method to stimulate gene clusters which are ‘silent’, ‘orphan’, and ‘cryptic’ for enhancing secondary metabolite expression without altering genes or causing the hereditable manipulation of microorganisms. Feng et al. [9] conducted an epigenetic manipulation of the aforementioned *Phomopsis asparagi* DHS-48 with the histone deacetylase (HDAC) inhibitor sodium butyrate and the DNA methyltransferase (DNMT) inhibitor 5-azacytidine (5-Aza). Based on the colony growth, dry biomass, HPLC, and ^1^H NMR analyses, the fungal chemical profile was significantly different compared to the control, and an optional modifier (50 µM sodium butyrate) was selected for the follow up fermentation. Two undescribed compounds, named phaseolorin J and phomoparagin D, along with three previously reported chromones and previously described cytochalasins, were isolated from the culture treated with sodium butyrate. Their structures, including absolute configurations, were elucidated using a combination of detailed HRESIMS, NMR, and ECD analyses and ^13^C NMR calculations. Phaseolorin J and phaseolorin J2 were found to be moderate immunosuppressants, inhibiting the proliferation of ConA (concanavalin A)-induced T and LPS (lipopolysaccharide)-induced B murine spleen lymphocytes. Additionally, phomoparagin D exhibited significant in vitro cytotoxicity against the human cancer cell lines Hela and HepG2.

Cai et al. [10] described two previously undescribed chlorinated metabolites, 8-chlorine-5-hydroxy-2,3-dimethyl-7-methoxychromone and 3,4-dichloro-1*H*-pyrrole-2,5-dione, together with eight known polyketides from the mangrove sediment-derived fungus Mollisia sp. SCSIO4140. X-ray single-crystal diffraction allowed the assignment of its absolute configuration of (4*S*, 5*S*, 13*R*, 14*R*, 17*R*, 18*R*, 21*R*)-stemphone C for the first time. 3,4-Dichloro-1*H*-pyrrole-2,5-dione and stemphone C showed different degrees of antimicrobial activity against several pathogenic fungi and bacteria, and antiproliferative activities against two human prostate cancer cell lines, PC-3 and 22Rv1. Furthermore, stemphone C was found to exhibit antiproliferative activity against two prostate cancer cell lines (PC-3 and 22Rv1) along with HepG2, WPMY-1, and MC3T3-E1. This compound reduced PC-3 cell colony formation, inducing apoptosis, and blocked the cell cycle at *S*-phase in a dose-dependent manner, revealing its use as a potential antiproliferative agent and a promising anti-prostate cancer agent.

Red yeast rice (*Monascus*-fermented rice, also called anka or koji) has been used as a natural food coloring additive and in traditional Chinese medicine since ancient times due to its ability to ease digestion and antiseptic effects. A novel strain *Monascus purpureus* wmd2424 was isolated from the mangrove in Chiayi Wetland. It was identified by colony culture morphology, microstructural characteristics, and partial sequence analysis of the β-tubulin gene fragments by Wu et al. [11]. The authors described the isolation of five previously unreported compounds, named monascuspurins A–E from the EtOAc extract of wmd2424 cultured in RGY medium. Of these, monascuspurins C–E exhibited mild antifungal activity against the tested *Aspergillus niger*, *Penicillium italicum*, *Candida albicans*, and *Saccharomyces cerevisiae*.

We would like to thank all the authors for their contribution to this Special Issue. The articles on the topic presented in this Special Issue revealed that mangrove ecosystems is especially attractive due to its abundant microbial communities, such as diverse strains of endophytic fungi from mangrove vegetation and sediments that produce various secondary metabolites with unusual skeletons. These compounds usually possess highly selective and specific biological activities with unique mechanisms of action, offering a promising prospect of mangrove ecosystems to serve as an unlimited reservoir for lead discovery and drug development. Biogenetic manipulation to stimulate silent or cryptic biosynthetic genes offers an effective strategy for mining untapped natural products from microorganisms. Nevertheless, the biosynthesis of most of the natural products derived from mangrove-associated microorganisms and their corresponding biosynthetic gene clusters in the mentioned microbial strains remain elusive.
